# Examining assisted suicide and euthanasia through the lens of healthcare quality

**DOI:** 10.1007/s11845-023-03418-2

**Published:** 2023-06-10

**Authors:** Miriam Colleran, Anne M. Doherty

**Affiliations:** 1 St Brigid’s Hospice, Crotanstown, Kildare Ireland; 2 Naas Hospital, Co Kildare, Naas, Ireland; 3 Department of Psychiatry, University College Dublin, 63 Eccles Street, Dublin 7, Ireland; 4 Department of Liaison Psychiatry, Mater Misericordiae University Hospital, 63 Eccles Street, Dublin 7, Ireland

**Keywords:** Assisted suicide, Ethics, Euthanasia, Palliative care, Palliative medicine, Quality of healthcare

## Abstract

Many people on both sides of the debate to legalise physician-hastened death are motivated by compassion and a desire to provide better end of life care for others. Assisted dying may include euthanasia and/or assisted suicide (EAS). It is legal in some jurisdictions and under debate in others including Ireland. EAS is a complex, sensitive and can be an emotive issue; detailed and nuanced examination of the subject is needed. To enhance this discussion, we examine EAS through the lens of quality. In examining EAS from this stance, we consider the action, along with the outcomes, the impact of the outcomes from other jurisdictions with legalised EAS, alongside the risks and the balancing measures used, in addition to considering the intervention itself. Progressive expansion of eligibility for EAS has occurred over time in the Netherlands, Belgium and Canada. Given the complexity of assessing coercion, the risks to persons in vulnerable groups (including older persons, persons with mental health conditions and persons with disabilities), the progressive expansion of eligibility for EAS, the lack of safety and the undermining of suicide prevention strategies, the current law is most protective of persons in vulnerable groups in the interest of social justice. Person-centred and compassionate care needs be prioritised with greater access and equitable access to primary and specialist palliative care and mental health care for persons with incurable and terminal illnesses and support for caregivers allowing patients to die naturally with optimised symptom control.

## Introduction

In Ireland, the 2020 Dying with Dignity Bill proposed to legalise euthanasia and/or assisted suicide (EAS) [1].

The Bill was unsuccessful after a public consultation process, and recommendations were made instead which included the option of the further progression of the Bill to a special committee. The legal opinion stated:

*“The ambiguities and serious drafting errors in sections 7, 8, 9, 10 and 11 of the Bill, in their current form, contain flaws that could potentially also render them vulnerable to challenge before the courts. Sections 7, 8, 9, 10 and 11 could be collectively termed the ‘safeguarding provisions’ of the Bill. As discussed in the legal briefing, these safeguarding provisions contain ambiguities and errors that arise as a result of significant questions of policy not being addressed in the Bill* [1]*.”*

Different countries use different terminologies and sometimes the same words with different meanings. In the US, legal assisted dying refers to assisted suicide or physician prescribed hastened death. The doctor is indirectly involved by prescribing the medication that causes death, but the medication is self-administered. In Canada, where the term Medical Assistance in Dying (MAiD) is used, this includes both assisted suicide and euthanasia. Similarly, voluntary assisted dying (VAD) in Australia refers to both assisted suicide and euthanasia.

Media reports discuss both public support and concerns about assisted dying [2, 3] and include the formal campaigning for legalisation for assisted dying in a newspaper circulated in Ireland and the UK [3, 4]. But does the term mean the same thing to everyone in such an important topic? The newspaper campaign used the same term across two different jurisdictions where the proposed legislation differed. The proposed legislation in the UK was to legalise physician assisted suicide only. However, here in Ireland, the proposed legislation was to legalise both euthanasia and physician-assisted suicide.

An online survey by the UK All-Party Parliamentary Group for Dying Well in 2021, of 1032 persons aged 18 years and older, reported that only 43% of participants believed assisted dying meant giving persons with a prognosis of less than six months medication to lead to their death [5]. Forty-two percent believed that it gave the right to refuse life sustaining treatments and 10% believed that it meant the provision of hospice care to persons who are dying; and the remaining 5% of participants did not know. This shows that 57% of participants did not know that assisted dying meant deliberately hastening death, and indeed conflated it with existing measures already available. Only 43% of the adult participants were aware that they could refuse disease-modifying treatment and still receive palliation and symptom control. One-quarter (24%) believed they had the right to decline life sustaining therapy but by doing so that they would not be able to receive other forms of care [5].

This emphasises the importance of language and the need to avoid confusion in any discussions on this sensitive topic. The government of the Netherlands, where assisted suicide and euthanasia are legally practiced since 2002, uses these terms along physician-assisted suicide [6]*.* This is congruent with the descriptions of euthanasia and physician-assisted suicide discussed by Duncan and Jeffrey [7].

In Ireland, an adult with capacity has the right to decline treatment, including life-sustaining treatments. This is an important right. Furthermore, doctors are not required to give non-beneficial treatments. Likewise withdrawing non-beneficial treatments is also ethically appropriate. As recommended by Emanuel and Joffe [8], they advised using the term euthanasia to refer to active and voluntary euthanasia only so as to prevent confusion. Furthermore, Emanuel et al. [9] recommended avoiding the term passive euthanasia as it refers to the above rather than an intervention to hasten the person’s death.

Using the terms assisted dying or assisted death to refer to both assisted suicide only and also to physician-assisted suicide and physician-delivered euthanasia can be confusing and may lead to uncertainty and a risk of misunderstanding. To avoid this, in this article, we will use the term euthanasia or assisted suicide (EAS) to encompass both physician-assisted suicide and physician-delivered euthanasia.

## The intervention

When considering EAS through the lens of quality, the aim of the intervention is the death of the person to whom the medication is administered. There is a paucity of evidence regarding the mechanism of pharmacologically hastened deaths: there is a lack of post mortem evidence on the mechanism of the person’s death.

In order to understand the process, we must consider other deliberately pharmacologically induced deaths. While the aim of execution is very different to EAS, the pharmacological mechanism of death by lethal injection is the same. Jivot, an anaesthesiologist, was invited by a prisoner to witness his execution and to review his post-mortem findings and reported that the person undergoing execution appeared comfortable, without outward signs of distress. However, post mortem examination reports on persons who had died by lethal injection describe signs of pulmonary fluid. This was consistent with pre-mortem rather than post-mortem changes [10]. This suggests that persons undergoing death by lethal injection experience pulmonary oedema and may be aware or unaware of it: the witness impression of the comfort of the prisoner’s execution may not be congruent with the person’s experience [10, 11]. Campaigns to legalise EAS frequently focus on the avoidance of suffering, but the current evidence base is insufficient to prove that EAS itself is without suffering. Furthermore, Jivot’s report of pharmacologically induced death and pulmonary oedema needs further investigation, given that cardiac and non-cardiac pulmonary edema are associated with increasing shortness of breath, a raised respiratory rate and crepitations [12].

When considering introducing and altering medical interventions, we are guided by the principles of evidence-based medicine, considering the hierarchy of research including randomised controlled trials, meta-analyses, prospective and retrospective studies and case series. Changes to medical practice should be evidence-based and science-informed, then person-centred and individualised to the particular person. In case of EAS, there is a lack of evidence for the pathophysiological mechanism whereby persons die through either euthanasia or physician prescribed hastened death. This raises an ethical question: do we consider it acceptable to deliver this treatment with little consideration of the evidence? New treatments including immunotherapies and vaccines are extensively researched before introduced into the care of some persons with advanced, terminal illness but other persons with similar diseases are treated differently and given poorly evidenced medications to cause hastened deaths. Perhaps there is a need, in the spirit of open disclosure, to inform persons enquiring about EAS of its lack of evidence.

Unlike other interventions, EAS tends to be introduced secondary to politico-legal considerations rather than clinical research [13, 14]. It was noted by Davis, in a reply to an editorial in the BMJ, that this perpetuated the illusion that assisted dying had a proven, scientific basis [14].

Bill C-14 introduced MAiD for persons who were terminally ill in Canada [15]. Where countries have legalised forms of EAS, this is typically introduced for persons who are terminally ill. This may be later challenged as discrimination against others for whom it is not legal. This occurred in the case of Truchon and Gladu v. Canada and Québec in the Superior Court of Québec [16] which declared that the eligibility criterion of the person being at the end of their lives and of having a prognosis of fairly predictable natural death in federal MAiD legislation and in Québec’s Act Respecting End-of-Life Care respectively were unconstitutional. This ruling only pertained to Québec [15]. However, it was not challenged further in court but led to the introduction of bill C-7 which widened the eligibility to include a second system for persons who were not expected to die soon naturally [17].

### The aims of and indications for the intervention

The primary reasons cited for legalising EAS are the prevention and relief of suffering and the autonomy of the person [18]. Personal autonomy is highly valued in society, in healthcare, ethics and especially in Western culture. Here, in Ireland, competent adults have the right to refuse care, including life-sustaining treatments [19]. While campaigns to legalise EAS often focus on preventing painful deaths, concerns related to the loss of autonomy feature in the practice. In 2020, 7384 persons died by EAS in Canada; the reasons included loss of the ability to engage in meaningful activities (84.9%), loss of ability to perform activities of daily living (81.7%), inadequate control of pain (or concern for this in the future) (57.4%), loss of dignity (53.9%) and isolation or loneliness (18.6%) [20]. The 2021 Oregon DWDA report [21] described end of life worries and unease. The concerns of the 238 people who died were as follows: losing autonomy (222), being less able to engage in activities that made life pleasurable (*n* = 219), loss of dignity (162), being a burden on family, friends and carers (129), the loss of control of bodily functions (112), insufficient pain control (or a concern regarding future pain control) (64) and the financial impact of treatment [21].

When considering a person’s autonomy, Demeter (2021) reported that Mill’s harm principle dictated that the only reason authority could be exerted over another person against the latter’s own autonomy was to block damage or detriment being inflicted on others.

Compassion and sensitivity are key in considering the care of persons with terminal and life-limiting conditions. While symptoms can mostly be well controlled with timely and appropriate palliative care, some individuals experience more difficult symptoms. Access to adequate palliative care services is needed as there can be challenges or inequities in accessing palliative care. In the UK, a leader in palliative care, there were issues about the funding of palliative and end of life care with a reliance on charity funding. An estimated 118,000 persons per year in the UK die without access to palliative care, with minority and marginalised groups least likely to access it [22, 23]. A successful campaign has led to the right to palliative care in the UK, a right which necessitates designated healthcare funding (HospiceUK, 2017). In Canada only 15% of people who died in 2016–2017 had access to palliative homecare, and only 6% in long-term care had access to palliative care [24]. While Ireland, like the UK, has overall good access to end of life care; some regions (the midlands and northeast) currently are without access to a specialist palliative care unit.

The later phase of a person’s life, as they approach dying, is a natural phase of life. Health and social care planning for persons approaching the end of their lives needs to be an integrated part of accessible publicly funded health and social care. Where funding is available, there is a need for adequate skilled staff to provide care to the patient and loved ones/families. The author (MC) is familiar with clinical situations where a publicly funded homecare package was approved for a person but there was a lack or absence of carers to deliver it or access was delayed. Dr. Ira Byock, past President of the American Association for Hospice and Palliative Medicine, has promoted that care at the end of life should be a part of the continuum of the provision of the best healthcare achievable.

### The prevalence of persons dying by EAS

Where EAS is legalised, the frequency of death by EAS increases after legalisation [25, 26]. In Oregon, for example, there were 24 persons who received prescriptions of lethal doses of medications in 1998 with 16 deaths under the Death with Dignity Act (DWDA). In 2021, this rose to 383 prescriptions and 238 deaths.(20) Oregon removed the residency requirement this year, allowing persons to travel for assisted suicide there [26]. Other states with longer duration of EAS, have reported a progressive expansion of eligibility criteria and provision. Initial introductory plans to restrict EAS to persons with terminal illnesses and conditions were successfully challenged in Quebec, Canada and deemed unconstitutional. Essentially, once legalised for a particular group in society, it was discriminatory towards others (Truchon & Gladu V Canada and _2019) [16]. Eligibility broadened in the Netherlands to include mental illnesses, in Belgium to include children in 2014 and in the Netherlands newborns via the Groningen Protocol [27, 28]. The Belgian Society of Intensive Care Medicine published a statement paper on giving sedating medications to patients without a possibility of recovery with the intention of hastening their deaths*.* They discussed that this was not just acceptable but also appropriate [28]. In 2015, EAS accounted for 4.5% of deaths in the Netherlands, of which 93% were performed by a GP [29]. Where both medically assisted suicide and clinician-delivered euthanasia are legal, the latter is predominantly practiced [30, 31]. There are geographical variations even within jurisdictions: in the Netherlands, there is a 25-fold regional variation between the highest and lowest of three regions assessed [31].

## Risks

Risks of a process are a key concern with any new treatment or procedure from a quality of care perspective. With EAS, the most serious adverse outcome is the inappropriate death of a person. Other aspects to consider include the reporting of EAS deaths, the adherence to procedures and processes and the impact on others.

### Doctors and physician hastened death

Annually less than 1% of licenced doctors provide prescriptions for EAS in Oregon and Washington [9]. In Oregon, 133 doctors in 2021 prescribed lethal prescriptions of medications for assisted suicide: a range of 1–47 prescriptions per doctor with 238 persons dying under the bill [21]. Over three-quarters of doctors (77%) prescribed one or two prescriptions. The median duration of the therapeutic relationship between the doctor and the patient in Oregon had decreased from 12 weeks between 1998 and 2019 to 5 weeks in 2021. It was unknown whether or not a complication had occurred in 163 cases [21]

In Canada in 2020, 1274 healthcare professionals who delivered EAS were medical doctors and 71 were nurse practitioners [20]. A study of 247 residents in family medicine/general practice training reported 41% were willing to engage in EAS, 31% in MAID by prescription (assisted suicide) and 24% in euthanasia [25].

### Adherence to protocols

Assessments for EAS can be done by telehealth in Canada [30]. In 2017 in Colorado, 69 eligible persons with prognoses of six months or shorter were prescribed medication to pharmacologically induce death in 2017 [32]. In 9 of the 69 cases where people were prescribed medication for assisted suicide, the form from the prescribing doctors was not received. In the majority of cases (42), the consulting physician’s form was not [32]. A study in Belgium showed the underreporting of assisted deaths, with approximately half of deaths formally reported [33]. Controversy occurred in Canada when Delta Hospice Society, which received (over half) public funding refused to provide EAS: it was threatened with the withdrawal of funding [34]. There have been concerning individual reports too, including that of a man with a neurological condition with audio evidence of hospital staff offering EAS when he requested homecare [35].

## Balancing measures or safeguards

All medical interventions and procedures can have adverse outcomes or complications [9]. The greatest risk from EAS is irreversible: the risk of the inappropriate death of a person. Considering EAS from a quality perspective means considering the impact on people with terminal illnesses requesting EAS, and the impact on others also. There is an underacknowledged need for balancing measures or safeguards to prevent inappropriate deaths. The development of safeguards will require the examination of the impact of EAS on clinical practice, narratives and personal experiences, along with the experiences of potentially vulnerable groups including persons with disabilities, older persons and persons with mental illnesses.

Typically, the reporting of deaths by EAS is retrospective. Regarding reporting procedures and the ability to audit and assess practice, the practice regarding death certification for persons who die by EAS varies. Colombia requires prior approval by an independent committee for deaths by EAS [9]. In New Zealand, where EAS was introduced in 2021, it must be recorded on death certificates as the direct cause of death [36]. Similarly, in the Netherlands, the death of a person through euthanasia or assisted suicide is deemed not natural and must be included on the person’s death certificate [37]. Vermont, Washington State, Québec, and Belgium do not list EAS on the medical death certificate [38]. In Colorado, recording deaths from EAS are recorded as deaths from the underlying disease. Self-reporting by the clinicians of deaths by EAS, and associated underreporting of deaths by EAS is known to occur. Of the 208 identified deaths by EAS in the Flanders study [39], 66 deaths were without demand from the patient, over half of these cases involved persons aged 80 years of age or more. In the majority of these patients’ deaths, the person had either had a non-cancer diagnosis (67.5%) or died in hospital (67.1%) [39]. In one-fifth of these cases (22.1%), the discussion of EAS was held with the patient but not at the latter’s initiation.

The regional variation in the recording of assisted deaths on medical death certificates, the underreporting of deaths, posthumous assessment of the deaths and the focus on the mechanism of the death or underlying cause all contribute to inaccuracies in the data on EAS.

The wish to hasten death (WTHD) is a phenomenon reported in the literature. A safeguard or balancing measure for assisted death typically include a reflection period to enable the person to change his or her mind if wished. The TILDA study in Ireland, reported that 3.5% of the 8174 adults aged 50 years or more who were living in the community had expressed a wish to die [40]. Two years later, following treatment for depression, 72% of those no longer had a wish to die. The timeframes in EAS legislation are much shorter and the safeguards embedded in legislation to prevent the misuse of EAS progressively change and wane over time as occurred in Canada and the Netherlands.

In Canada, of the 9375 written applications for EAS in 2020, 2.5% (232) of the requests were withdrawn: due to change of minds (66.4%) or because palliative treatments were satisfactory (47.8%). Over a fifth of the withdrawn applications were just before the intervention when the person was asked to give their final consent for EAS [21]. The minimum of a ten-day period for rumination or contemplation, a balancing measure or safeguard, was removed in bill C7 in Canada for persons whose death is anticipated. That means that where a person’s death is predictable, the individual no longer needs to wait before receiving EAS [17]. This is in contrast to the scientific research on the transient nature of wish to die for the majority of persons who experience it [40]. Under Canada’s changes and expansion of EAS eligibility to persons whose death is not fairly predictable*,* a parallel system is in operation where the eligibility appraisal must take at least ninety days [17]. Ireland’s halted 2020 Dying with Dignity Bill, had a minimum contemplation period of 14 days unless the person’s prognosis was considered to be less than a month, where it was to be 6 days [1]. The balancing measure of reflection time, to allow for change of mind if wished, in Canada can be decreased depending on estimated prognosis, as outlined in Sect. 3.1 (i) of the C7 Act [17]. A study of patients with cancer who received EAS between 2017 and 2019 in Alberta, Canada, reported 87.6% received EAS within three months of requesting, when their symptom burden was highest. Two-thirds had EAS carried out the same month they requested it. During the last month of life, the majority of patients (close to 60%) had a high symptom burden [41].

In practice, initial legislation for EAS usually restricts it to persons who are terminally ill. This was successfully challenged in Canada as being excessively restrictive and discriminatory [42]. Prognostication, however, is complex and inaccurate especially the further a person is from their anticipated death. Clinically, we prognosticate in terms of years, months, long months, short months, weeks, long weeks, short weeks, days and day by day. Prognostication is particularly challenging when a person has a non-cancer diagnosis, and predictions are frequently inaccurate [43]. In the case of the proposed Dying with Dignity bill in Ireland, a specific duration of prognosis is not required [1]. The eligibility criteria refers to person being *“terminally ill”* [1]. However, the term terminal is not defined in the proposed bill. Persons with stage IV cancers, Gold stage IV COPD advanced pulmonary fibrosis, dementia and end-stage cardiac failure all have incurable illnesses, but the prognoses can vary greatly. This bill was not confined to persons who might have prognoses of short months, weeks or less.

At the Winter Conference 2021 of the College of Psychiatrists of Ireland, Professor Sonu Gaind explained that Canada was the first country in the world to introduce EAS for persons who had not received any disease-modifying treatment [44]. In other jurisdictions, a person is considered eligible for EAS only after treatment has failed. Ireland’s unsuccessful 2020 Dying with Dignity Bill echoed the Canadian bill in that the person was not required to have received any treatment: the person was required to be *“fully informed”* of treatment options [45]. As such, the unsuccessful bill included persons with unaddressed, unmodified and untreated symptoms rather than persons with uncontrolled symptoms solely.

More recently, media reports are raising concerns about the practice of EAS in Canada, especially regarding the euthanasia of persons with disabilities who have inadequate social supports or inappropriate housing [46, 48]. Ableism is perhaps less discussed than other forms of discrimination such as racism, sectarianism and gender bias. Para-olympian and House of Lords member Baroness Tanni Grey-Thompson, a wheelchair user, has shared her experiences of people saying to her that they would no longer want to be alive if they were like her [47]*.* Media reports have highlighted persons with disabilities utilising EAS in Canada due to a lack of social supports [48, 50]. This raises the question: is EAS truly an autonomous decision if the person is not empowered with the means to live?

## Vulnerable groups

### Decision-making capacity

A person must have decision-making capacity to be able to make a life-changing decision. In the unsuccessful 2020 Dying with Dignity bill, Section 10(4), the person only had to retain the information for a short period of time to be considered to have capacity [1]. This is in contradiction of Section 9(6) of the bill that the person can change their mind at any time, and indeed a contradiction of the functional assessment of capacity set out in the Assisted Decision-Making (Capacity) Act (2015) [49]. The overall mechanism for the assessment of capacity was poorly set out, and presumably was to be predicated upon the future enactment of the Assisted Decision-Making Act (2015). It did not consider who would be qualified to assess capacity in this context, nor how a difference of opinion between the two assessing physicians might be addressed.

### Coercion and elder abuse

Section 9.3© of the proposed Dying with Dignity bill [1] stated that both doctors needed to be satisfied that the person seeking EAS had “*a clear and settled intention to end his or her own life which has been reached voluntarily, on an informed basis and without coercion or duress.”* However, assessing coercion is very challenging. Professor David Kissane, an Australian psychiatrist specialising in psycho-oncology and palliative care, submitted to the “*Inquiry into the provisions of the Voluntary Assisted Dying Bill 2021”* in New South Wales. He shared four stories of de-identified patients who had requested or experienced an assisted death. In the first patient story, the oncologist patient’s symptoms were under managed by the oncologist who became primarily focussed on the process of EAS distracting away from symptom control. Another de-identified patient, was reported to have been pressurised to EAS by an adult child home on holidays. This caused bereavement challenges for his wife who had anticipated having longer time with him. In a further case, while the oncologist referred the patient to a psychiatrist, although the patient’s family were encouraging the patient to request EAS. The psychiatrist diagnosed clinical depression and commenced treatment with effect [50].

A recent study of the quality of end-of-life care in eighty-one countries, ranked the United Kingdom and Ireland first and second respectively: neither of which has legislated for EAS. Countries with EAS were ranked as follows: Australia ranked fourth, Canada twenty-second, Belgium twenty-sixth and Columbia forty-second [51]. Assisted suicide is legal in some states in the US, which ranked forty-third. New Zealand ranked twelfth in the study, which was performed prior to the legalisation of EAS with the enactment of the End of Life Choices Act [52]. Canada’s ranking in the 2015 Quality of Death index prior to the introduction of EAS was eleventh.

### Risks to persons who may be vulnerable

In a quality framework, an intervention is considered in terms of the outcome, the benefits and the risks, changes that can occur from introducing the action and the balancing measures taken to counteract possible adverse results from the changed intervention. It can be difficult to balance the needs of the individual with those of others in society who may be impacted by legislating for the few. Viewing an intervention from the perspective of quality makes it necessary to assess the risks from the intervention. The unintended risks posed by legislation for EAS to persons who may be vulnerable including older persons, persons with disabilities and people with mental health conditions needs to be considered. These risks need to be weighed alongside arguments in favour of EAS from the perspective of autonomy.

Elder abuse or the abuse of older persons is a unfortunately a well-described experience internationally [53, 54]. The pooled prevalence rate of elder abuse was 15.7% from a meta-analysis of 52 studies [54]. Allowing for the lack of data from low- and middle-income countries, approximately 1 in 6 older persons experience abuse. In Ireland, 2592 referrals were made to the HSE Elder Abuse service in 2014. Public health nursing (21%) was the most likely to refer persons for concerns about experiencing elder abuse, hospitals, community HSE health and social care professionals and GPs (15%, 9% and 7%, respectively) [59, 53]. An Australian report on the abuse of older persons showed that abuse was most frequently carried out by a son or daughter (either biological or adopted), friend, or spouse or partner [55].

In 2019, Devandas-Aguilar, the then UN Rapporteur for Disabilities in an End of Mission statement, expressed concerns regarding the impact of Canadian legalisation for EAS on persons with disabilities. Furthermore, Devandas-Aguilar discussed the absence of a structure to ensure that persons with disabilities met the criteria for EAS had meaningful alternatives to it. She also reported that she was informed of allegations that persons who were disabilities were being pushed towards medically assisted dying and of the underreporting of their deaths by EAS [56].

This, however, predated the new C-7 bill which extend EAS to include persons with disabilities on the basis of disability alone [17]. Disparities exist in society. Quinn, De Schutter and Mahler, Special Rapponteurs with the Special Procedures of the UN Human Rights Council commented that a much greater proportion of persons with disabilities are impoverished than able bodied persons and on the risk of the former choosing a hastened death from desperation [57]*.*

In Ireland, the Report on the Joint Committee on Justice on Scrutiny of the Dying with Dignity Bill 2020, some submissions by older persons discussed that having the bill under discussion seemed to indicate that society considered them, namely, older persons, of *“little value* [58]*.”*

In the states in the US where EAS is legal, persons undergoing EAS are typically white, educated and have insurance [9].

However, in the European countries where EAS is available and Canada, a wider picture emerges. It is now legal for doctors in the Netherlands to perform EAS on persons with dementia who had previously consented to EAS and to prevent them from resisting [59]. In one prominent Dutch case, a woman with dementia who had completed an advance directive five years beforehand that she wished to die at a time of her own choosing, she objected at that particular time but was sedated for EAS by a sedative in her coffee and was held down by her family at the doctor’s instruction [60, 61]. The supreme court decided that laws were not broken and that if a patient can no longer give consent, the doctor did not have to take an exact interpretation of the advance directive if the situation did not match [61].

This poses significant challenges from an ethical perspective. Sedation to prevent a person resisting EAS is a form of chemical restraint. At a minimum, it is very worrisome but it risks being misused and harmful. The stated aims of EAS are to support self-determination and autonomy and to minimise distress. It is difficult to reconcile this with sedating the person to prevent the person from resisting the actual act of EAS, and functionally removes the person’s ability to withdraw consent. This may be interpreted as a form of discrimination against persons with dementia. Such practice alters the dynamic of the doctor-patient relationship from a collaborative engagement with informed patient decision-making, to the patient being disempowered with the decision making, control and timing of the act of EAS entirely devolved to the clinician. Brown described dehumanisation as an onward practice [62]. This might be considered a step in that process of dehumanisation.

### The impact on persons with mental illness

EAS is available for persons with primary diagnosis of mental illness in the Netherlands [63] and will be available for people with mental illnesses in Canada from March 17^th^ 2023 [64]. Given that people with mental illness are overrepresented among the population of people who die by suicide, it is difficult to envisage how permitting assisted suicide for people with mental illnesses might not be seen to be undermining of public health efforts to reduce the suicide rates of many countries [65]. It is unclear that the risk factors for ‘traditional’ suicide differ to any significant degree from that for assisted suicide where this is lawful for people with primary mental illnesses [66]. It is greatly at variance with the aims and the ethos of the ‘zero suicide’ movement [67]. This does not consider the vulnerabilities of people with co-morbid terminal illnesses and mental illness. Depression is common in people experiencing pain, and as such pain may be regarded as a risk factor for suicidal thoughts and actions [68].

In considering EAS from a quality perspective, it is important to examine the long-term impact where it has been introduced. One essential question is: did the safeguards or balancing measures work in practice? What, if any, was the impact on total number of self-instituted deaths [69]. “Self-initiated death” includes deaths by non-assisted suicide and EAS [69]. Posner made the claim that by making dying by suicide easier that it might result in lower numbers of overall deaths by suicide [70]*.* Posner purported that a remarkable number of people were incorrectly told that they had terminal illnesses or given erroneously short prognoses [70]. He postulated that if physician assisted dying was less expensive than a person dying by suicide that they would favour physician assisted suicide. The involvement of a doctor would decrease the numbers of persons from dying by suicide impetuously as they awaited the medical appointment and furthermore, by the doctor diagnosing and managing mental health issues [70]*.* Posner’s theory [70] implies that persons died by suicide because EAS was not available to them. Girma and Paton summarising Posner’s Theory suggested that persons avail of assisted dying instead of dying by self-instituted suicide [71]*.* However, they also noted that although a person can definitely anticipate what they might do in the future and a person who assumed that they would want to die by suicide in the future might not actually want to when the situation arises [71]*.* Posner’s theory suggests that by permitting EAS, the numbers of non-assisted suicides should decrease correspondingly. Jones compared EAS in the Belgium and the Netherlands with similar countries similar in language, culture and location [69]. Non-assisted deaths did not decrease greater with respect to similar matched countries. Furthermore the overall numbers of self-instituted deaths increased and there was no reduction in non-assisted deaths with respect to matched neighbouring European countries not practising hastened deaths [69]. A recent systematic review of the relationship between suicide and EAS demonstrated no such decrease and that in older women had a sharp increase in numbers of self-instituted deaths [72]. This is concerning as older women have higher rates of depressive illnesses, and it is unknown if they accessed appropriate assessment and treatments. Similarly evidence from the USA of some states that legalised assisted suicide shows an increase in total suicides or self-instituted deaths especially for older persons and women [69]. Posner’s proposal is not supported by current evidence [69, 70].

The impact of legalisation of EAS is complex. Practising suicide prevention for some persons and legalising a hastened death for others is contradictory. It suggests that some lives are considered less worthy of suicide prevention measures than others, and that persons are viewed differently because of having a terminal illness or disability.

## Conclusion

Dame Cicely Saunders, the founder of the current hospice and palliative medicine movement wrote:

‘You matter because you are you. You matter to the last moment of your life, and we will do all we can to help you not only to die peacefully, but also to live until you die.’ [73]

Many societies are trying to debate how to compassionately support persons with terminal illnesses. Despite the emphasis on evidence-based practice and quality improvement in healthcare, there is a lack of empirical research regarding EAS. Progressive expansion has occurred over time in the Netherlands, Belgium and Canada. There is a legitimate concern that where there are challenging and pressurised demands on healthcare resources, the right to die will become the duty to die. Given the complexity of assessing coercion, the risks to persons in vulnerable groups (including older persons, persons with mental health conditions and persons with disabilities), the progressive expansion of eligibility for EAS, the lack of safety and the undermining of suicide prevention strategies, the current law is most protective of persons in vulnerable groups in the interest of social justice. Current legalisation in Ireland does prevent some members of society from accessing EAS. However, person-centred and compassionate care needs be prioritised with greater access and equitable access to primary and specialist palliative care and mental health care for persons with incurable and terminal illnesses and support for caregivers allowing patients to die naturally with optimised symptom control.
